# The Experience of the Salim Yusuf Emerging Leaders Programme: A Journey Beyond Borders

**DOI:** 10.5334/gh.1381

**Published:** 2024-12-17

**Authors:** Vagner Madrini Junior, Vernice R. Peterson, Patrick Ngassa Piotie, Swagata Kumar Sahoo, Sonali Munot, Rochelle Regina Cruz, Daniele Rodolico, James Ayodele Ogunmodede, Gonzalo Rodriguez

**Affiliations:** 1 Albert Einstein Hospital, São Paulo, BR; 2 Heart Institute, São Paulo, BR; 3 University of the Witwatersrand, ZA; 4 University of Pretoria, ZA; 5 Resolve to Save Lives, US; 6 University of Sydney, AU; 7 Cardiovascular Institute of Cardinal Santos Medical Center, PH; 8 University of Pisa, IT; 9 Univesity of Ilorin, NG; 10 Hospital Universitario Fundación Favaloro, AR

**Keywords:** Emerging Leaders, World Heart Federation, Global Health, Collaboration

Participating in the Salim Yusuf Emerging Leaders Programme was an exceptionally enriching experience that went far beyond the scope of project development. Celebrating its 10th anniversary this year, the program was hosted in Mombasa, Kenya—a location imbued with cultural and historical significance that served as a framework for the event that would follow. The initiative convened 25 emerging leaders from around the world, each bringing distinct perspectives and expertise, to address one of the foremost challenges in cardiovascular health. This year’s theme centered on the Single Pill Combination (SPC) strategy, an innovative approach aimed at improving medication adherence and reducing the global burden of cardiovascular disease. The task we were given—to design an SPC-based project that could be implemented with funding from the World Heart Federation (WHF)—was both challenging and inspiring. Our projects were expected not only to address medication adherence but also to consider the socio-economic and healthcare contexts of different regions. This required us to go beyond theoretical solutions and develop actionable, realistic approaches to address the gaps that avert utilization of this evidenced based recommendation. By grounding our ideas in practicality and adaptability, we sought to create projects that could truly make a difference. The WHF’s support, mentoring, and commitment to funding these projects added an invaluable layer of accountability, encouraging us to think carefully about the real-world implications of our work.

A crucial aspect of the program was the emphasis on collaborative teamwork. Working closely with peers from around the world was an eye-opening experience that required us to set aside our own preconceived notions and be open to diverse perspectives. Each participant brought a unique viewpoint shaped by their cultural and healthcare backgrounds, creating a rich environment for exchange. We quickly engaged onto the fact that effective solutions should encompass this comprehensive approach. Working in such a collaborative atmosphere fostered a spirit of unity and reminded us that healthcare challenges are shared globally, even if they manifest differently across regions.

Holding the program in Africa added a significant depth to the experience. Africa is a continent that faces both immense healthcare challenges and extraordinary opportunities for innovation and progress. Being in Kenya allowed us to witness firsthand the unique health system challenges faced by African nations, as well as the determination and resilience of local healthcare providers. The program underscored that cross-border partnerships can play a vital role in addressing these challenges, even for countries with different settings. In addition, it highlighted the importance of supporting and empowering regions with high disease burdens but limited resources, which ties in well with the African proverb: ‘If you want to go fast go alone; but if you want to go far, go together’.

This is where international organizations such as the WHF and initiatives like the Emerging Leaders Program can play a relevant role, fostering the organization of collective efforts, promoting global and local leadership, and collaborating in the design of plans and strategies to improve access, availability, and utilization of resources to impact health systems. It underscores that global health equity relies on ensuring that all communities have access to essential and high-quality healthcare. For this, support through shared knowledge, resources, and policies to address disparities is crucial, especially in high-burden and underserved regions. This experience highlights the importance of a global perspective in health leadership, encouraging us to step beyond our own contexts and approach health issues as interconnected challenges, with a strong belief that implementation science, cooperation, and hard work can truly make a difference.

The Salim Yusuf Emerging Leaders Programme represents far more than a project-based initiative. It opens the door to a mindset of collective inquiry, integration, and cooperation aimed at making a positive impact on global health. The project we developed is just one part of a much broader experience filled with learning, cultural exchange, and collaborative problem-solving. This journey has been an intensive training that empowered us with the skills, connections, and motivation to contribute to global health in meaningful ways, inspiring each of us to continue working toward a more equitable, interconnected, and healthy world.

